# Endovascular hypothermia after cardiac arrest in a Chilean ICU

**DOI:** 10.1186/cc13684

**Published:** 2014-03-17

**Authors:** M Canitrot, S Ugarte

**Affiliations:** 1 INDISA Clinic, Santiago, Chile

## Introduction

For more than a decade, mild hypothermia has been a standard for enhancing the neurological prognosis in comatose survivors of cardiac arrest (CA). Despite this, in our country there are still few centers that apply hypothermia post CA regularly. We describe a Chilean experience with endovascular hypothermia post CA, in three ICUs of the same university clinical center [1-3].

## Methods

A descriptive cohort study. All surviving comatose patients after CA were included, and underwent endovascular hypothermia management according to protocol. CoolGard™ internal cooling equipment was used. Variables: delay between CA and hypothermia (34°C), time in hypothermia, complications, ventilatory and hemodynamic management. Main outcome measures: mortality and neurological follow-up to 6 months with Glasgow Outcome Score Extended (GOSE).

## Results

**Figure 1:**
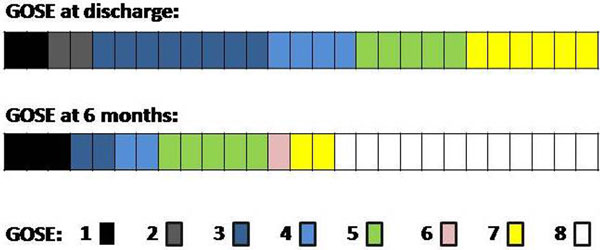


Twenty-seven patients were managed in the ICU post CA (18 outpatients). Twenty-four were men (89%), mean age 33.5 ± 19 years (16 to 76). Delay was considered (time between CA and achieving target temperature of 34°C): median 10.5 ± 3.3 hours (3 to 18 hours). Hypothermic maintenance (from when it reaches 34°C until it returns to 36°C): 24 ± 18 hours (24 to 48 hours). Complications of hypothermia: five hypokalemia (18.5%), three ventricular arrhythmias (11%), one vein thrombosis (3.7%). There were no deaths during hypothermia. Hospital mortality was two cases (7.4%). At 6 months it was three (11%). Neurological outcome at discharge and 6 months are shown in Figure 1. Good outcome (GOSE 5 to 8) occurred in 11 patients (40%) at discharge. Good outcome at 6 months occurred in 20 patients (74%).

## Conclusion

Our series shows a low mortality and a very good neurological outcome. There was no mortality or severe complications associated with endovascular hypothermia. It is a safe and feasible technique implemented in Latin American critical care units. Even the delay in achieving the objective of hypothermia is very long.
